# Tokyo-172 BCG Vaccination Complications, Taiwan

**DOI:** 10.3201/eid1509.081336

**Published:** 2009-09

**Authors:** Ruwen Jou, Wei-Lun Huang, Wei-Ju Su

**Affiliations:** Author affiliation: Taiwan Centers for Disease Control, Taipei, Taiwan

**Keywords:** Tuberculosis and other mycobacteria, lymphadentitis, osteitis, osteomyelitis vaccination, BCG vaccination complications, Taiwan, letter

**To the Editor**: BCG (*Mycobacterium bovis* BCG) is a vaccine for preventing childhood tuberculosis (TB), especially military and meningeal TB. Because Taiwan has an annual TB incidence rate of ≈70 cases/100,000 persons, the National Immunization Program has included neonatal BCG vaccination since 1965. The coverage rate has remained at 97% since 2001. According to the Taiwan Tuberculosis Registry, the median rate of TB infections diagnosed in patients <10 years of age during 2005–2007 was 0.39% (60 cases) (*1*). The risk of developing childhood extrapulmonary TB without lung involvement is highest among children <5 years of age.

In 1965, the World Health Organization registered freeze-dried Tokyo-172 seed lot as an international reference vaccine strain (*2*). Tokyo-172 BCG is currently used in Taiwan, Japan, and South Korea. The vaccine is recommended as less reactogenic. Because intradermal injection is recognized as a more effective BCG administration route (*3*), it is practiced in Taiwan, while a multiple puncture method is used in Japan and South Korea. In addition, 10% BCG (Danish strain) vaccinations for infants are administrated intracutaneously in South Korea (*4*). Although BCG is effective in preventing progressive primary TB, adverse reactions to the vaccine do occur. A systemic review of adverse reactions has been established in Japan (*5*) but not in South Korea (*4*) and Taiwan (*6*).

During 1951–2004, a total of 39 cases of severe adverse vaccine reactions were reported in Japan, with an incidence rate of 0.182 cases of reactions/million vaccinations. Of the 39 cases, 27 patients (69.2%) had bone and joint involvement, and 13 (33.3%) had primary immunodeficiency (*4*). One patient had both complications. The BCG vaccine was initially produced in Taiwan using Pasteur-1173 P2 strain (0.025 mg/0.1mL) and changed to the less reactogenic Tokyo-172 strain (0.05 mg/0.1 mL) in 1979. From 1998 through 2007, 14 patients applied for compensation through the vaccine injury compensation program for BCG-caused adverse reaction, and 6 claims were confirmed. Of the 6 confirmed BCG complications cases, 5 patients had humeral or sternal osteomyelitis and 1 patient died from a disseminated BCG infection. Accordingly, in 2002–2006 the risk for BCG osteitis/osteomyelitis and disseminated BCG infection was 3.68 and 0.9 per million, respectively (*6*). Incidence of severe complications was higher than that documented in Japan.

Because Taiwan lacks diagnosis and postmarketing surveillance system, BCG-related complications might be underreported. As part of initiating a comprehensive adverse events surveillance, a laboratory program to differentiate *M*. *bovis* BCG from other species of the *M*. *tuberculosis* complex was established to monitor local adverse events (injection-site abscess, lymphadenitis) and severe complications (suppurative lymphadentitis, BCG osteitis/osteomyelitis, and disseminated BCG infection [BCGitis]) among vaccinated children.

During 2005–2007, 19 clinical specimens (6 biopsy samples and 13 bacterial isolates) of suspected BCG-infection childhood TB cases were sent to the Taiwan Centers for Disease Control. To differentiate among *M. tuberculosis*, *M. bovis*, and *M. bovis* BCG, DNA samples were initially screened using a GenoType kit (Hain Lifescience GmbH, Nehren, Germany), multiplex PCR (*7*), and pncA sequencing (*8*). In addition, spoligotyping was performed with a commercial kit (Isogen Bioscience BV, Maarssen, the Netherlands). An additional multiplex PCR (*9*) was used to differentiate vaccine strains. Medical charts were reviewed to determine sites of involvement and severity of complications.

Of the 19 patients, 1 (5.3%), 2 (10.5%), 15 (78.9%), and 1 (5.3%) were infected with *M. tuberculosis* complex, *M. tuberculosis*, *M. bovis* BCG, and *M. abscessus*, respectively. All identified *M. bovis* BCG isolates had the same spoligotype as the Tokyo-172 vaccine strain. The median age of BCG-related complication patients was 2 years (range 1–9 years), and the male:female ratio was 1.5. Of the 15 *M*. *bovis* BCG–infected patients, 14 had extrapulmonary sites of involvement, including 8 bone and joint, 3 suppurative lymphadenitis in axillary lymph node, 2 subcutaneous abscess away from the injection site, 1 injection-site abscess, and 1 disseminated BCGitis (Table).

**Table:** Characteristics *Mycobacterium* *bovis* BCG complication cases, Taiwan, 2005–2007*

Patient no.	Sex/age at diagnosis, y	Year reported	Specimen	Diagnosis and site of involvement
1	F/2	2005	Biopsy sample	BCG osteitis/osteomyelitis, right ankle
2	M/1	2005	Bacterial isolate	Subcutaneous abscess, left anterior chest wall
3	M/2	2005	Bacterial isolate	Severe combined immunodeficiency, disseminated BCGitis
4	M/9	2005	Bacterial isolate	Suppurative lymphadenitis
5	F/1	2005	Bacterial isolate	Injection-site abscess
6	M/1	2005	Biopsy sample	Suppurative lymphadenitis
7	M/2	2006	Bacterial isolate	BCG osteitis/osteomyelitis, right distal femoris
8	M/2	2006	Bacterial isolate	BCG osteitis/osteomyelitis
9	F/1	2006	Bacterial isolate	BCG osteitis/osteomyelitis, left distal femoris
10	F/1	2006	Bacterial isolate	BCG osteitis/osteomyelitis, left distal radius
11	F/2	2007	Bacterial isolate	BCG osteitis/osteomyelitis, right knee
12	M/1	2007	Bacterial isolate	Subcutaneous abscess, left wrist
13	M/2	2007	Biopsy sample	BCG osteitis/osteomyelitis, right ankle
14	F/1	2007	Bacterial isolate	Suppurative lymphadentitis
15	M/2	2007	Bacterial isolate	BCG osteitis/osteomyelitis, left proximal tibia

*BCGitis, disseminated BCG infection.

According to an international survey, the estimated rate of osteitis/osteomyelitis is 1–700/1,000,000 vaccinated newborns or infants with different strain-derived BCG (*10*). In Taiwan, the estimated incidence of BCG osteitis/osteomyelitis was 12.9 cases (8/621,853; 95% confidence interval 4–21.8) per million vaccinations during 2005–2007. Previously reported complications of BCG osteitis/osteomyelitis related to Tokyo-172 strain might be underestimated. The association between administration route and osteitis/osteomyelitis with Tokyo-172 BCG remains obscure. The potency and safety of BCG prepared from Tokyo-172 stain are under reevaluation as one of the action plans of National TB Program. The stability and quality control of vaccines strain, production processes, and intradermal injection techniques are being reappraised.

Furthermore, a policy of enhanced childhood TB surveillance was implemented in 2007, and clinicians were advised to send clinical specimens to the Centers for Disease Control in Taiwan for differential diagnosis of *M. bovis* BCG for patients <5 years of age. In particular, suspected childhood TB patients without an identifiable TB contact and with normal immune status were subjected to further investigations. Multidisciplinary management, including enhanced laboratory diagnosis of atypical bony lesions in infants and children, is recommended for any suspected TB infection. Once BCG-related infection is confirmed, medical treatment has to be consistent.
